# Sedentary Behaviour in Swiss Children and Adolescents: Disentangling Associations with the Perceived and Objectively Measured Environment

**DOI:** 10.3390/ijerph15050918

**Published:** 2018-05-04

**Authors:** Bettina Bringolf-Isler, Kees de Hoogh, Christian Schindler, Bengt Kayser, L. Suzanne Suggs, Alain Dössegger, Nicole Probst-Hensch, the SOPHYA Study Group

**Affiliations:** 1 Swiss Tropical and Public Health Institute, Socinstrasse 57, 4051 Basel, Switzerland; c.dehoogh@swisstph.ch (K.d.H.); christian.schindler@swisstph.ch (C.S.); nicole.probst@swisstph.ch (N.P.-H.); 2 University of Basel, Petersplatz 1, 4051 Basel, Switzerland; 3 Institute of Sport Sciences, University of Lausanne, 1015 Lausanne, Switzerland; bengt.kayser@unil.ch; 4 Institute of Public Communication, University della Svizzera italiana, Via G. Buffi 13, 6900 Lugano, Switzerland; suzanne.suggs@usi.ch; 5 Swiss Federal Institute of Sport Magglingen SFISM, 2532 Magglingen, Switzerland; alain.doessegger@baspo.admin.ch

**Keywords:** sedentary behaviour, accelerometer, home environment, perceived environment, GIS, objective environment, children, adolescents, neighbourhood, social environment, urbanicity, walkability, public health

## Abstract

Identifying correlates of sedentary behaviour across all levels of the ecological model and understanding their interrelations is a promising method to plan effective interventions. The present study examined whether the objectively assessed and the perceived neighbourhood are associated with children’s sedentary behaviour time (SBT). A comprehensive set of factors at different levels of influence across the ecological model were taken into account and analysed for mediating and modifying effects. Analyses were based on 1306 children and adolescents (6–16 years) participating in the population-based SOPHYA-study. Accelerometers were used to assess SBT, the perceived environment was examined by a validated parental questionnaire, and objective environmental data were allocated using GIS (ArcMap 10.2, Esri, Redlands, CA, USA) for each family’s residential address. A high perceived safety was associated with less SBT. Boys, those whose residential neighbourhood was characterized by dead ends in urban areas, a low main street density in the neighbourhood of children and greenness were less likely to exhibit SBT. The association of the objective environment with the respective parental perceptions was low and no significant mediating effect was found for the perceived environment. We conclude for land-use planning to reduce sedentary behaviour objective environments should be complemented with efforts to increase parental sense of security.

## 1. Introduction

There is a growing body of evidence that sedentary behaviour (SB) may be a distinct risk factor for type II diabetes, cardiovascular disease, cancer and all-cause mortality in adults, independent of physical activity (PA) [1,2,3]. In children, the evidence on the association between sedentary behaviour and health outcomes is less obvious [4]. Lifestyle related diseases are rare at this age, but chronic diseases are thought to develop over the course of life and have roots in childhood [5]. There is great interest in identifying correlates of SB in children as it tracks from childhood into adulthood [6] and intervening at an early age may be an effective strategy [7]. Prolonged, uninterrupted sitting rather than total sedentary behaviour time (SBT) had negative health effects in adults [8,9]. Yet, children typically accumulated sedentary bouts of relatively short duration [4,10,11]. In addition, the interpretation of the existing literature is compromised by the different assessment of SB. Most studies in children are based on self or proxy reports of screen time, which is a poor indicator of SB [4,11,12,13]. Objective measures from accelerometers provide more precise data on SB but the use of such devices in large samples often lacks necessary financing.

To understand determinants of PA ecological models [14], considering the multiple levels of influence (individual, social environmental and policy), and their interrelations have been developed. These models are also applicable for SB-research [15] although SB and PA may not be associated with each other and be influenced by different factors [16,17]. Interventions on the environmental and the policy level are promising as they are modifiable factors, reach a large proportion of the population, and have bigger potential for long-term impacts than individual interventions. Barriers in the neighbourhood can be objective (e.g., GIS based) or perceived. Both can have an impact on children’s SB [18,19]. The objective environment could also influence parental perceptions and thus have an indirect impact on children’s SBT. To understand their separate contributions and interrelations, both, objective and subjective factors must be studied [20] in the context of pathway analyses, that consider demographic sub-groups [21,22]. For the association between SBT and neighbourhood factors differences by age and sex were described [22,23,24]. Some studies reported associations between children’s overall or screen time with the social [23,25] and the home environment [20] as well as the perceived [26] and the objectively assessed neighbourhood factors [22,25,27], while others could identify correlates only in boys [28] or not at all [29]. Although a recent review [7] concluded that determinants of SBT were found at the individual, interpersonal, environmental and policy level, only few studies have examined a comprehensive set of factors at different levels of influence.

Therefore, the aim of this study was to test whether the objectively assessed neighbourhood is associated with children’s SBT and whether this association is mediated by the perceived environment. It also aimed to assess potential moderating effects by individual and social characteristics.

## 2. Materials and Methods

### 2.1. Study Population and Setting

For the analyses, all children born between 1998 and 2007 and living in Switzerland were eligible. They were recruited based on national inhabitant registry data. The SOPHYA-study [30,31] consisted of two parts: First a computer-aided telephone interview (CATI) about sport behaviour [32] was conducted by a professional field research institute (LINK Institute, Lucerne, Switzerland). 3113 families (85% participation rate) were interviewed between February 2013 and February 2015. During this interview, families were asked whether they could be contacted for a second assessment using accelerometers. The study period of this second part took place from December 2013 to June 2015. The study sample for this present study consists of families participating in both parts. Participants’ ages 6 to 11 years were categorized as “children” and ages 12 to 16 were categorized as “adolescents”. All parents and adolescents gave written informed consent for their participation. The study was conducted in accordance with the Declaration of Helsinki, and the protocol was approved by the ethics committee of Basel (147/13).

### 2.2. Sedentary Time

ST was assessed using model GT1M or GT3X accelerometers (ActiGraph, Pensacola, FL, USA). For these devices, the uniaxial output is compatible [33]. After a first instruction by phone the participants received the accelerometer and the user manual by postal mail. Children and adolescents were instructed to wear the accelerometer for seven consecutive days fixed on the right waist and to remove it for water activities and during sleeping hours. After the measurement week, the devices had to be sent back in a prepaid envelope. For the download and the data reduction, ActiLife 6.2 software (ActiGraph) was used. Epoch length was set at 15 s and non-wearing time was defined as a period of 60 min of consecutive 0 counts/min (cpm). The cut-point for SB was defined as an intensity of less than 100 cpm [34]. Mean total SBT was calculated separately for weekdays and weekend days by summing up sedentary minutes and dividing them by the number of measurement days. Prolonged sedentary bouts were defined as periods of at least 10 consecutive minutes below 100 cpm without any tolerance time within a bout [10,11]. All minutes spent in such bouts were summed up and a mean per weekday or weekend day was calculated. On weekdays time before 12 noon was excluded, assuming, that children spent this time at school and not in the neighbourhood.

### 2.3. Perceived Neighbourhood Data

The perceived neighbourhood was assessed in a parental questionnaire using the scores of the Australian CLAN study (Children Living in Active Neighborhoods) [34]. Scores were computed for perceptions of road safety (three items), incivilities (four items) and personal safety of the child or adolescent (five items). All scores were based on single items with a five-point scale ranging from strong disagreement (scored as −2) to strong agreement (scored as 2). The single questions are listed in Table 2. In a test-retest-analysis [35] the items reached reliability of 0.41 to 0.81. In the same test conducted with 40 Swiss children 54% of the questions had a kappa of r > 0.6 (range 0.34–0.77). The valences of individual question scores were configured such that a higher score in the road safety perception denotes greater perceived danger; higher incivilities score denotes greater incivility; and greater personal safety score indicates greater perceived safetyA previously used question about access to parks and playgrounds [36] was added to the CLAN-study battery. The questionnaire was sent to the participants and returned by mail together with the accelerometer.

### 2.4. Objective Neighbourhood Data

The objective neighbourhood was assessed at the individual level using ArcMap 10.2 (Esri, Redlands, CA, USA). The neighbourhood attributes were used previously [22,37] and cover the same four aspects of the environment as those for the perceived environment. Objective road safety was represented by the main street density (m/200 m radius) and derived from Swiss Topo Vector 25 (swisstopo, Wabern, Switzerland) [38]. The crime rate per 100,000 inhabitants [39] was used as a measure of aesthetics and incivilities. Four variables were used to assess personal safety: The walkability index, the number of dead ends within a 200 m radius [38], the number of schoolchildren within 100 m [39] and the distance to the nearest bus, tram or railway station [38]. The walkability was calculated at a 1 × 1 resolution and was based on the z-scores of the residential density, land use mix and street connectivity [37]. For land use mix, the land use types ‘residential’, ‘industrial or commercial units’ and ‘sport and leisure facilities’ were extracted from Corine landcover 2006 [40] and street connectivity was defined as the number of intersections extracted from Swiss Topo Vector 25. For greenness we extracted the satellite-derived normalised difference vegetation index (NDVI) for the year 2014 which is based on satellite data [41]. The Swiss neighbourhood index of socioeconomic position (SEP) [42] was included as a possible moderator [22]. The Swiss SEP is a score from 1 to 10 whereby scores of 5 and less denotes low SEP. It has been validated and shown to be related to all-cause mortality [42]. The language regions and the classification of areas into “urban”, “agglomeration” and “rural” referred to the definition of the Federal Office of Statistics [43].

### 2.5. Statistical Analyses

**Figure 1:**
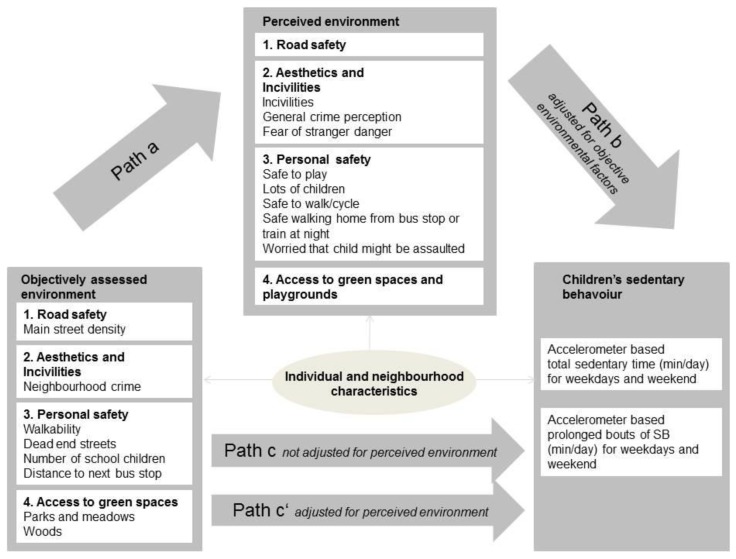
Mediation effects of the perceived environment on the associations between children’s sedentary time and the objectively assessed environment. c coefficients: estimates of the associations between each item of the objective environment and children’s time spent sedentary. c’ coefficients: estimates of the associations between the items of the objective environment and children’s time spent sedentary, adjusted for the items of the perceived environment. a Coefficients: estimates of the associations between items of the objective environment and the items of the perceived environment. b Coefficients: estimates of the associations between the items of the perceived environment and children’s time spent sedentary, adjusted for the items of the for the objective environment. The c’-path describes the direct effect of the objective environment on children’s sedentarytime, the a-path × b-path the possible indirect effect. The total effect c = c’ + a × b.

The mean SBT and prolonged sedentary bouts (dependent variable) by sociodemographic and environmental factors were calculated using linear regression models adjusted for age, sex and mean daily accelerometer time. Associations between time of SB and different predictor variables were assessed using linear regression models. First, the time variables were regressed against sociodemographics (age, sex, household income) neighbourhood characteristics (SEP, language region, residential area), and measurement-related factors (season, accelerometer time, device). For all subsequent analyses involving objective environmental characteristics and single items or scores of the perceived environment questionnaire, the time variables were subject to a square root transformation, to remove skewness from the residuals. Mediating effects of the perceived environment on the association between the objectively assessed neighbourhood and was assessed by applying the product-of-coefficient method (Figure 1). The following associations were assessed: (i) between the different items of the objective environment and the children’s time of SBT (c-path); (ii) between items of the objective environment and the respective scores of the perceived environment (a-path); and (iii) between scores of the perceived environment and children’s time of SBT (b-path). Finally, associations between the items of the objective environment adjusted for the respective item of the perceived environment were estimated (c’-path). All analyses were adjusted for sociodemographics, neighbourhood characteristics and measurement-related factors. The statistical significance of the mediating effect was assessed using the Sobel-Test [44] provided that the c-path was significant. To understand whether associations of SBT with the perceived and the objectively assessed environment were modified by sociodemographics or neighbourhood characteristics, interaction analyses were performed.

Missing values in factor variables were removed by introducing an additional category, and single imputation was used to remove missing values in covariates. Participants with missing values in the dependent variables were excluded from analyses. All analyses were conducted with STATA 14.0 (Statacorp, Lakeway Drive, TX, USA, 2015).

## 3. Results

### 3.1. Study Population

A random sample of 2032 families was contacted for the accelerometer part of the SOPHYA-study. Of these families, 421 (20.7%) could not be included in the present analyses because they revoked their interest, 39 (1.9%) because of technical problems, and 252 (12.4%) because they did not fulfil the criterion of at least 3 weekdays with valid accelerometer data of 10 h and one weekend day of 8 h. An additional, 14 (0.7%) children were excluded because of missing questionnaire data. The final sample consisted of 1306 (64.3%) children and adolescents ages 6 to 16.

### 3.2. Total Sedentary Time and Prolonged Sedentary Bouts

Mean duration of SBT during leisure time was 318.2 min/day on weekdays and 446.0 min/day on the weekend days. On average 77.7 min/day (weekday) and 108.2 (weekend) were spent in prolonged sedentary bouts. There was a high correlation between the percentage of total SBT and the percentage of time spent in prolonged sedentary-bouts per mean accelerometer time (r = 0.8). SBT increased with age and girls accumulated more sedentary minutes than boys (Table 1). Mean sedentary time was higher among children and adolescents living in the French and Italian speaking part of Switzerland than among those from the German speaking part, both on weekdays and weekends. However, the differences reached statistical significance only on the weekdays. The lowest amount of sedentary minutes was accumulated in the summer and the highest in the winter. 

### 3.3. The Perceived Environment

Parental perceptions of the environment were generally more favourable if families lived in high SEP areas, in the German speaking part or in non-urban areas (data not shown). Of the calculated scores of the perceived environment, the personal safety-score was the only one showing an inverse association with SBT (Table 2). 

All other scores showed associations in the expected direction, albeit not statistically significant. In subgroup analyses, among children, but not adolescents, having a poor perception of aesthetics and incivilities by parents showed a significant positive association with SBT, with average changes in SBT per unit increment of the respective score of 0.06 (95%-CI: 0.01; 0.10) respectively −0.02 (95%-CI: −0.08; 0.04) (*p* for interaction = 0.04). Results were comparable for prolonged sedentary bouts (Table S1), although the association with perceived safety became statistically insignificant.

### 3.4. The Objectively Assessed Environment

There were geographic differences in objectively assessed environmental factors: The road safety, the crime rate and the green spaces had more favourable levels in high SEP areas, in the German speaking part and in non-urban areas (data not shown). The correlation between the scores for the perceived environment and its respective factors of the objectively assessed environment were weak to non-existent (r = −0.04 to 0.3) (Table S2).

### 3.5. Mediating and Modifying Effects on SBT on Weekday

Of the items representing the objective environment, only the amount of green space was associated with SBT on the weekdays (see c-path Table 3). This association remained statistically significant after adjustment for perceived access to play areas. Although some associations between the items of the objectively assessed environment and the respective scores of the perceived environment were found (a-path), not evidence was found for a mediating role of the perceived environment in the observed associations between objective environmental characteristics and SBT. For example, the associations of objectively assessed road safety and aesthetics/incivilities factors with respective perceived factors did not translate into a mediating effect of environmental perception. The association of perceived safety with SBT was independent of adjustment for objective environmental variables. This was not true when considering prolonged sedentary bouts as outcome (Table S3); proximity to the public transport was inversely associated with SBT, the effect was independent of perceived personal safety, and the independent effect of perceived personal safety was not statistically significant. 

**Figure 2:**
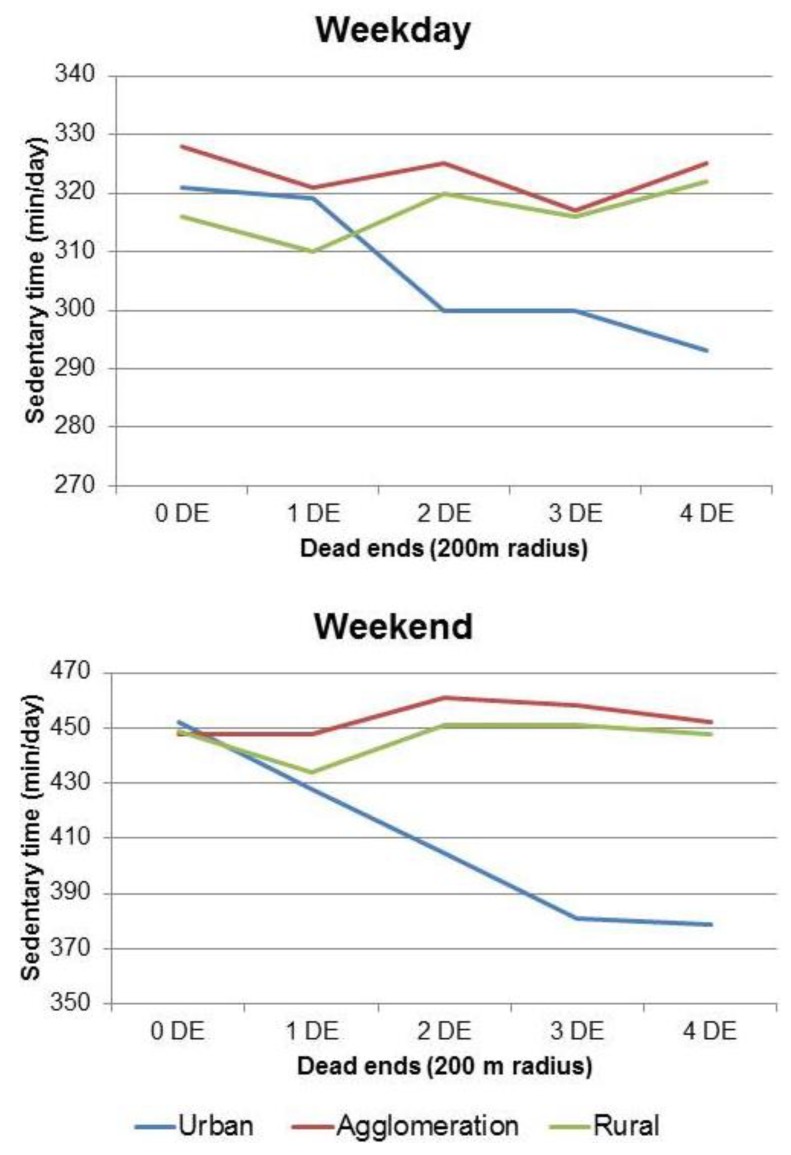
Association between sedentary time and dead ends by urbanicity on weekdays and the weekend. Adjusted for age, sex and accelerometer time. The graph includes only associations based on 5 children and more per category. P for interaction on weekdays: *p* = 0.003 and p for interaction in the weekend: *p* = 0.002.

In subgroup analysis, urbanicity was the most important effect modifier for the association between the objectively assessed environment and SBT (c-path): SBT on weekdays was significantly lower if urban children lived in areas with a lot of dead ends (Figure 2), while there was no such association among children from rural areas.

### 3.6. Mediating and Modifying Effects on SBT in the Weekend

**Figure 3:**
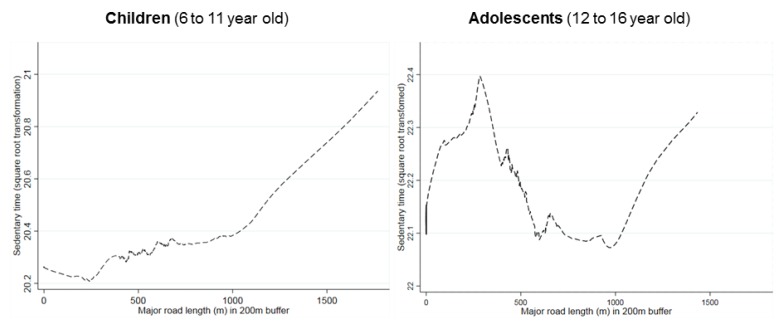
Association between time spent sedentary in the weekend and main street density by age-group. P for interaction = 0.01, Coefficient for children: 0.03 (0.0; 0.07); Coefficient for adolescents: −0.2 (−0.7; 0.2).

Total SBT during the weekend was not associated with greenness or any of the objectively measured parameters in the total sample (Table 4). Again, no significant mediation effect by the scores of the perceived environment was found. Consistent with the observation for weekdays, the association of a high number of dead ends was associated with less SBT for children in urban areas (Figure 2). The main street density was associated with SBT of children but not of adolescents (Figure 3).

## 4. Discussion

We aimed to assess whether the objectively assessed neighbourhood was associated with children’s total SBT and prolonged sedentary bouts and whether these associations are mediated by the perceived environment taking into account a comprehensive set of factors at different levels of influence across the ecological model. Total SBT and prolonged bouts were highly correlated, and their determinants were quite similar. SBT was lower in children and boys, if perceived personal safety was high, if the main street density in the neighbourhood of children was low, and in urban areas if there was a high number of dead ends in the neighbourhood. On weekdays SBT was lower if GIS-based greenness was high. The associations were coherent in direction for weekdays and the weekend.There was no indication for perceived environmental quality to have a mediating role in associations between SBT and objective environmental characteristics.

It is a matter of scientific debate whether total SBT or prolonged bouts should be measured [10,11]. In the present study the association of both variables with various environmental factors was similar. Consistent with previous studies, the mean sedentary bouts were short and prolonged bouts were rare [10,11]. We therefore conclude that overall SBT is a good proxy for prolonged bouts in children.

Several sociodemographic factors were significantly associated with ST. Boys were less sedentary than girls although several studies showed that boys spend more time with screen activities [45]. This confirms that screen activities are only one aspect of SB and should not be used as a synonym [46]. Especially on weekdays, SBT was lower among German speaking participants than among their counterparts from the other language regions. This is consistent with findings of PA behaviour [47,48,49] and could not be explained by differences in the objectively assessed environment [48]. Another study showed that even within the bilingual city of Biel-Bienne (Switzerland), German speaking children commute to school more often than the French speaking children [36] although the built environment and the school system is the same. Also policies are comparable between the language regions: In Switzerland all children normally have three physical education lessons per week at school. It might thus be that cultural background has an important role. Cultural factors were found to explain a significant proportion of the variance in safety concerns [50] and, in the present study, parents from the German speaking part perceived their environment more positively.

Previous studies concluded that, in order to understand associations and pathways through which the environment has an impact on PA and SB, both, objective and subjective environmental factors and their interrelation must be assessed in various subgroups. [21,51,52]. The present analyses followed these recommendations. In line with previous findings [53,54], objective environmental characteristics and their perceived equivalents were only weakly correlated (see also Table S2) and showed independent associations with SBT. Parental perceptions did not explain associations between the objectively assessed environment and SBT. Among the scores representing different aspects of parental perceptions only the perceived safety score was significantly associated with ST. This confirms previous findings, concluding that parents who worry about their children’s safety may reduce their outdoors activities, resulting in higher SBT [55]. Negatively perceived aesthetics was significantly associated with SBT in children but not in adolescents. Yet, the scores for aesthetics were highly skewed and, in general, parental concerns were low compared to an Australian sample answering to the same questionnaire [35]. This resulted in a low variance and reduced the power for detecting associations.

Green space was the only objectively assessed factor that was significantly associated with less SBT in the total sample. Two previous studies reported similar findings [22,56], while two other ones did not [29,57]. The association was stronger on weekdays than the weekend, possibly because on weekends families have time for excursions. On the contrary, the association between the main street density with children’s SBT was only significant in the weekend. It might be that there are different pathways explaining how the environment is associated with ST. Environmental factors may either be supportive for or barriers against an active behaviour and these factors may apply differently to different people.

The strongest effect modifier identified was urbanicity. The association between the objective environment and SBT varied by whether or not children lived in urban environments. While dead ends, an indicator for low transit traffic and often used as meeting/play areas, were associated with less SBT in urban children, this was not true for non-urban ones. This is consistent with a previous study, showing that the main street density was significantly associated with time spent playing outdoors in areas with a medium to high population density but not in those with a low one [58]. It seems that children living in urban areas are more dependent on having play areas included in land use planning, whereas unhindered access to play areas seems to be more common in rural areas. In our study the walkability index was only associated with SBT in urban children, yet only on the weekend. However, the walkability index was developed for adults and a Belgian study concluded that street connectivity can improve walking in adults but may result in less activity in children [59]. Furthermore, the walkability score was developed for urban areas in the US and might be less meaningful in rural areas or in Europe [22,60].

The associations between the environment and SBT seem small at a first glance, but considering the ubiquitous exposure to these environmental factors, their impact on SBT at a population level may be considerable. In addition, children and adolescents do not spend all their time in their neighbourhood, and this results in a measurement error and thus in underestimates of associations. While a limitation, it is still a valuable approach as studies that included GPS showed that children tend to spend most of their time close to their home [61,62]. A further limitation is the cross-sectional design making it impossible to draw any conclusions about causality. Finally, accelerometers underestimate intensity of activities spent cycling and swimming. The study has several strengths. First, it included objective measures for SB using validated cut-points [46]. Second the recruitment was based on a large random sample of families drawn from registry data, thus avoiding clusters of schools or regions. Third, objective environmental factors and scores reflecting their perceptions among parents were included in the models and associations were analyzsed taking into account multiple levels of influence across the ecological model. This made it possible to assess potential pathways and variations in observed associations across different study subgroups. 

## 5. Conclusions

This study shows that associations between SBT and the environment differ by subgroups, particularly between urban and rural areas and when the environment is assessed objectively or by self-report. Therefore, interventions and conclusions for land use planning should be adapted to the target region and the target population. For instance, while greenness was associated with less SBT across all subgroups, children in urban areas appear to especially profit from activity friendly meeting areas. The pathway analysis revealed that the objectively assessed environment has little influence on how parents perceive their neighbourhood. Therefore, interventions promoting an active behaviour and less SBT should not only focus on structural changes but also include efforts to increase parental sense of security. Future studies analyzsing the association between SBT and the neighbourhood need to include both, the perceived and the objective environment.

## Figures and Tables

**Table 1:** Differences in sedentary time according to individual and neighbourhood characteristics.

Characteristics	Category	*n* (%)	Weekdays		Weekend	
			Total Time Spent Sedentary	Time Spent Sedentary in Bouts of at Least 10 min	Total Time Spent Sedentary	Time Spent Sedentary in Bouts of at Least 10 min
			Mean Min/Day (SE)	Mean Min/Day (SE)	Mean Min/Day (SE)	Mean Min/Day (SE)
Overall	all	1306 (100)	318.2 (2.0)	77.7 (1.4)	446.0 (2.2)	108.2 (2.0)
Age	6 to 11 years (ref.)	838 (64.2)	299.7 (1.4)	62.9 (1.4)	419.6 (2.4)	87.7 (2.3)
	12 to 16 years	468 (35.8)	351.5 (1.9) ***	104.4 (1.9) ***	493.3 (3.1) ***	144.7 (3.0) ***
Sex	Boy (ref.)	671 (51.4)	308.7 (1.4)	72.2 (1.5)	432.0 (2.5)	100.6 (2.5)
	Girl	635 (48.6)	328.3 (1.4) ***	83.6 (1.5) ***	460.9 (2.5) ***	**116.1 (2.5)**
Household income	<6000 CHF (ref.)	272 (20.8)	315.8 (2.1)	74.2 (2.3)	450.0 (3.9)	106.4 (3.9)
	6000 to 9000 CHF	409 (31.3)	318.6 (1.7)	79.5 (1.9)	445.6 (3.2)	107.8 (3.1)
	9000 and more	475 (36.3)	319.0 (1.6)	77.9 (1.7)	446.1 (2.9)	110.4 (2.9)
	No information	150 (11.5)	318.1 (5.0)	74.8 (5.3)	439.5 (5.2)	104.8 (5.2)
Swiss socioeconomic neighbourhood index	Low (score 1 to 5) (ref.)	612 (46.9)	317.9 (1.5)	77.2(1.5)	442.8 (2.6)	105.7 (2.6)
	High (score 6 to 10)	694 (53.1)	318.4 (1.4)	78.3 (1.4)	448.8 (2.4)	110.2 (2.4)
Language region	German (ref.)	907 (69.5)	315.1 (1.2)	76.7 (1.3)	444.3 (2.1)	107.8 (2.1)
	French	250 (19.1)	323.6 (2.2) ***	80.7 (2.4)	449.1 (4.0)	107.7 (4.0)
	Italian	149 (11.4)	328.0 (3.0) ***	78.9 (3.1)	451.6 (5.2)	110.7 (5.2)
Residential area	Urban (ref.)	250 (19.1)	318.7 (2.3)	77.7 (2.4)	438.0 (5.4)	109.3 (4.2)
	Agglomeration	633 (48.5)	319.7 (1.4)	79.3 (1.5)	449.5 (3.4)	108.8 (2.6)
	Rural area	423 (32.4)	315.9 (1.8)	75.6 (1.8)	445.5 (4.1)	106.4 (3.1)
Season ^1^ of measurement	Spring (ref.)	376 (28.8)	318.9/1.9)	80.4 (2.0)	441.3 (3.4)	105.5 (3.4)
	Summer	180 (13.8)	308.3 (2.7) ***	76.0 (2.8)	431.4 (4.7)	103.00 (4.8)
	Autumn	322 (24.7)	318.6 (2.0)	76.6 (2.1)	444.3 (3.5)	109.8 (3.6)
	Winter	428 (32.8)	321.5 (1.7)	77.0 (1.8)	457.6 (3.1) ***	111.4 (3.1)

*** *p*-value ≤ 0.001 compared to reference. Adjusted for age, sex and accelerometer time. ^1^ Spring: March–May; Summer: June–August; Autumn: September–November; Winter: December–February.

**Table 2:** Independent associations of the perceived neighbourhood ^1^ with sedentary time on weekdays and the weekend.

Parental Perception of Neighbourhood	“Agree” or “Strongly Agree” %	Increase of SBT ^2^ per Increase of the Perceived Environment Score	
		Weekend Coeff. (95% CI) ^3^	Weekend Day Coeff. (95% CI) ^3^
1. Road safety score of −6 to +6 based on: four 5-point items scored from −2 to +2:		0.02 (−0.00; 0.03)	0.01 (−0.01; 0.04)
There are major barriers to walking/cycling in my local neighbourhood that make it hard for my child to get from place to place (e.g., freeways, major roads)	22.0	0.02 (−0.02; 0.07)	0.02 (−0.05; 0.08)
There is heavy traffic in our local streets.	33.5	0.04 (0.00; 0.08)	0.05 (−0.02; 0.11)
Road safety is a concern in our area.	15.6	0.05 (0.00; 0.10)	0.03 (−0.05; 0.11)
2. Aesthetics and incivilities score of −8 to +8 based on: four 5-point items scored from −2 to +2:		0.01 (−0.01; 0.04)	0.03 (−0.01; 0.06)
My neighbourhood is generally free from litter, rubbish, and graffiti.	91.9	−0.03 (−0.09; 0.04)	−0.04 (−0.14; 0.05)
There is a high crime rate in our neighbourhood	1.9	0.04 (−0.03; 0.12)	0.09 (−0.02; 0.21)
I am worried about troublemakers hanging around my neighbourhood.	3.5	0.00 (−0.07; 0.07)	0.02 (−0.09; 0.12)
Stranger danger is a concern of mine	16.6	0.03 (−0.02; 0.08)	0.04 (−0.04; 0.12)
3. Personal safety score of −10 to +10 based on: five 5-point items scored from −2 to +2:		−0.02 (−0.04; 0.00) **	−0.04 (−0.07; −0.02) ***
It is safe for my child to play or hang out in the street outside our house.	81.4	−0.07 (−0.13; −0.02) **	−0.16 (−0.24; −0.08) ***
Lots of children play or hang out in our street.	57.7	−0.03 (−0.07; 0.02)	−0.07 (−0.13; 0.00) *
My neighbourhood is safe for my child to walk/cycle around the block alone in the daytime.	83.7	−0.03 (−0.10; 0.03)	−0.08 (−0.18; 0.01)
My child would be safe walking home from a bus stop or train at night.	51.1	−0.03 (−0.08; 0.02)	−0.07 (−0.14; 0.01)
I am worried that my child might be assaulted when out alone in our neighbourhood.	6.83	0.09 (0.03; 0.15) **	0.15 (0.06; 0.24) ***
4. Access to parks and playgrounds: 5-point item (from −2 to +2):			
My child can play on a playground, park, or other public places (play street, schoolyard) in its neighbourhood without supervision.	82.9	−0.03 (−0.08; 0.02)	−0.11 (−0.02; −0.19) *

* *p*-value ≤ 0.05; ** *p*-value ≤ 0.01; *** *p*-value ≤ 0.001. ^1^ All items had a five-point scale from strongly disagree (scored as −2) to strongly agree (scored as 2). ^2^ SB time was transformed using the square root. ^3^ Adjusted for age, sex, household income, socioeconomic neighbourhood index (SEP), language region, urbanicity, season, accelerometer time and device model. For road safety and aesthetics a higher score denotes less favourable environments and for the personal safety and access to parks and playgrounds a higher score denotes a more favourable environment.

**Table 3:** Mediation of the associations between the objectively assessed environment and children’s sedentary time on weekdays by the perceived environment.

Objectively Assessed Neighbourhood	Unit	Total Effect	Direct Effect	Indirect Effect			Significance of Mediation ^1^
		c-Path All Values for * 1000 Coeff (95% CI)	c’-Path All Values for * 1000 Coeff (95% CI)	a-Path All Values for * 1000 Coeff (95% CI)	b-Path Coeff. (95% CI)	ab-Path All Values for * 1000 Coeff. (SE)	
Road safety							
Main street density	(m/200 m buffer)	0.0 (−0.2; 0.2)	−0.0 (−0.2; 0.2)	2.3 (1.8; 2.8) ***	0.02 (−0.00; 0.03)	0.04 (0.02)	-
Aesthetics and incivilities							
Crime rate	n/100,000 inhabitants	−0.8 (−2.4; 0.9)	−0.9 (−2.6; 8.0)	10.8 (6.9; 14.7) ***	0.02 (−0.01; 0.04)	0.16 (0.14)	-
Personal safety							
Walkability	z-score (1000 m)	14.6 (−4.6; 33.7)	13.7 (−5.4; 32.8)	−43.5 (−108.8; 21.8)	−0.02 (−0.04; −0.00) *	0.8 (0.7)	-
Dead ends	n/200 m buffer	1.4 (−47.9; 50.7)	4.6 (−44.7; 53.8)	158.3 (−9.3; 325.9)	−0.02 (−0.04; −0.00) *	−3.1 (2.1)	-
Number of school children	n/100 m^2^	0.3 (−0.6; 1.1)	2.0 (−9.9; 5.8)	2.3 (−0.6; 5.2)	−0.02 (−0.04;−0.00) *	−0.05 (0.04)	-
Distance to public transport	in m	−0.0 (−0.2; 0.1)	−0.0 (−0.2; 0.1)	0.3 (−0.2: 0.8)	−0.02 (−0.04; −0.04) *	−0.006 (0.005)	-
Access to playgrounds							
Green space (NDVI)	score/1000 m buffer	−637.3 (−1214.6; −60.1) *	−625.1 (−1203; −47.3) *	450.9 (−144.6; 1046.3)	−0.03(−0.08 0.03)	−0.01 (0.01)	Non significant

* *p*-value ≤ 0.05; *** *p*-value ≤ 0.001. ^1^ The significance of the mediation was only tested if the total effect was statistically significant. SB time was transformed using the square root. All analyses adjusted for age, sex, household income, socioeconomic neighbourhood index, language region, urbanicity, season and accelerometer time. c: coefficients: estimates of the associations between each item of the objective environment and children’s time spent sedentary, e.g., main street density on time spent sedentary (square root transformation) on a weekday. c’: coefficients: estimates of the associations between the items of the objective environment and children’s time spent sedentary, adjusted for the items of the perceived environment (mediator), e.g., main street density on time spent sedentary (square root transformation) on a weekday, adjusted for the road safety score for parental perceptions. a: coefficients: estimates of the associations between items of the objective environment and the items of the perceived environment, e.g., main street density and the read safety score for parental perceptions. b: coefficients: estimates of the associations between the items of the perceived environment and children’s time spent sedentary, adjusted for the items of the for the objective environment, e.g., the road safety score for parental perceptions on time spent sedentary (square root transformation) on a weekday, adjusted for the main street density. The c’-path describes the direct effect of the objective environment on children’s sedentary time, the a-path × b-path the possible indirect effect (see also Figure 1). The total effect c = c’ + a × b.

**Table 4:** Mediation of the associations between the objectively assessed environment and children’s SB in the weekend by the perceived environment.

Objectively Assessed Neighbourhood	Unit	Total Effect	Direct Effect	Indirect Effect			Total Effect
		c-Path All Values for * 1000 Coeff. (95% CI)	c’-Path All Values for * 1000 Coeff. (95% CI)	a-Path All Values for * 1000 Coeff. (95% CI)	b-Path All Values for * 1000 Coeff (95% CI)	ab-Path All Values for * 1000 Coeff. (SE)	
Road safety							
Main street density	(m/200 m buffer)	0.1 (−0.1; 0.4)	0.1 (−0.2; 0.4)	2.3 (1.8; 2.9) ***	0.1 (−0.02; 0.04)	0.02 (0.03)	-
Aesthetics and incivilities							
Crime rate	n/100,000 inhabitants	−0.4 (−2.9; 2.1)	−0.7 (−3.3; 1.8)	11.1 (7.2; 14.9) ***	0.03 (−0.01; 0.06)	0.3 (0.2)	-
Personal safety							
Walkability	z-score (1000 m)	−22.4 (−51.5; 6.6))	−24.4 (−53.4; 4.5)	−44.7 (109.9; 20.6)	−0.04 (−0.07; −0.02) ***	2.0 (1.6)	-
Dead ends	n/200 m buffer	−54.1 (−128.8; 20.6)	−47.1 (−121.6; 27.3)	160.4 (−6.9; 327.99	−0.04 (−0.07 (−0.02) ***	−6.9 (4.2)	-
Number of school children	n/100 m^2^	−0.3 (−1.6: 1.0)	−0.1 (−12.0; 11.8)	2.2 (−0.6; 5.1)	−0.04 (−0.07: −0.02) ***	−0.1 (0.07)	-
Distance to public transport	In m	0.0 (−0.1; 0.3)	0.1 (−0.2; 0.3)	0.3 (−0.2; 0.8)	−0.04 (−0.07; −0.02) ***	−0.01 (0.01)	-
Access to playgrounds							
Green space (NDVI)	Score/1000 m Buffer	−344.1 (−1222.9; 534.6)	−294.7 (−1172.1 (582.8)	455.5 (−139; 1050.9)	−0.11 (−0.19; −0.03) **	−49.5 (37.9)	-

* *p*-value ≤ 0.05; ** *p*-value ≤ 0.01; *** *p*-value ≤ 0.001. ^1^ The significance of the mediation was only tested if the total effect was statistically significant. SB time was transformed using the square root. All analyses adjusted for age, sex, household income, socioeconomic neighbourhood index, language region, urbanicity, season and accelerometer time. c: coefficients: estimates of the associations between each item of the objective environment and children’s time spent sedentary, e.g., main street density on time spent sedentary (square root transformation) on a weekday. c’: coefficients: estimates of the associations between the items of the objective environment and children’s time spent sedentary, adjusted for the items of the perceived environment, e.g., main street density on time spent sedentary (square root transformation) on a weekday, adjusted for the road safety score for parental perceptions. a: coefficients: estimates of the associations between items of the objective environment and the items of the perceived environment, e.g., main street density and the read safety score for parental perceptions. b: coefficients: estimates of the associations between the items of the perceived environment and children’s time spent sedentary, adjusted for the items of the for the objective environment, e.g., the road safety score for parental perceptions on time spent sedentary (square root transformation) on a weekday, adjusted for the main street density. The c’-path describes the direct effect of the objective environment on children’s sedentary time, the a-path × b-path the possible indirect effect (see also Figure 1). The total effect c= c’ + a × b.

## Supplementary Materials

The following are available online at http://www.mdpi.com/1660-4601/15/5/918/s1, Table S1: Independent association of the perceived neighbourhood with sedentary behaviour in bouts of at least 10 min, Table S2: Correlation between the perceived environment and the respective objective measures, Table S3: Mediation effects of the perceived environment on the associations between the objectively assessed environment and children’s prolonged sedentary bouts on weekdays (square root transformation), Table S4: Mediation effects of the perceived environment on the associations between the objectively assessed environment and children’s prolonged sedentary bouts in the weekend.
