# Supplementary material for: Sedentary Behaviour in Swiss Children and Adolescents: Disentangling Associations with the Perceived and Objectively Measured Environment
Source: Int J Environ Res Public Health. 2018 May 4;15(5):918. doi: 10.3390/ijerph15050918 (PMC5981957; doi:10.3390/ijerph15050918)
Supplement: Supplementary file 1 [file ijerph-15-00918-s001.pdf]

Table S1. Independent association of the perceived neighbourhood [1] with sedentary behaviour in bouts of at least 10 minutes.

	"Agree"or "strongly agree"in %	Increase of SB [2] per increase of the perceived environment score	
		**Weekend Coeff. (95% CI) [3]**	**Weekend day Coeff. (95% CI) [3]**
**1.Road safety** score of -6 to +6 based on: three 5 point items scored from −2 to +2:		0.02 (−0.02; 0.06)	0.00 (−0.0; 0.05)
There are major barriers to walking/cycling in my local neighbourhood that that make it hard for my child to get from place to place (e.g., freeways, major roads)	22.0	0.02 (-0.08; 0.11)	0.00 (−0.1; 0.13)
There is heavy traffic in our local streets.	33.5	0.05 (−0.04; 0.13)	0.00 (−0.1; 0.12)
Road safety is a concern in our area.	15.6	0.07 (−0.04; 0.17)	0.03 (−0.1; 0.18)
**2. Aesthetics and incivilities** score of -8 to +8 based on: four 5 point items scored from −2 to +2:		0.03 (-0.02; 0.07)	0.00 (−0.0; 0.07)
My neighbourhood is generally free from litter, rubbish, and graffiti.	91.9	−0.11 (−0.24; 0.02)	−0.04 (−0.2; 0.14)
There is a high crime rate in our neighbourhood	1.9	0.07 (−0.08; 0.23)	0.04 (−0.1; 0.28)
I am worried about troublemakers hanging around my neighbourhood.	3.5	−0.02 (−0.16; 0.12)	−0.09 (−0.3; 0.12)
Stranger danger is a concern of mine	16.6	0.03 (−0.08; 0.13)	−0.02 (−0.1; 0.13)
**3.Personal safety** score of -10 to +10 based on: five 5 point items scored from −2 to +2:		-0.02 (-0.05; 0.01)	−0.04 (−0.0; 0.01)
It is safe for my child to play or hang out in the street outside our house.	81.4	**−0.14 (0.25; −0.02)***	**−0.17 (−0.32; −0.01)***
Lots of children play or hang out in our street.	57.7	0.00 (−0.09; 0.08)	−0.06 (−0.1; 0.07)
My neighbourhood is safe for my child to walk/cycle around the block alone in the daytime.	83.7	0.00 (−0.13; 0.13)	−0.03 (−0.2; 0.15)
My child would be safe walking home from a bus stop or train at night.	51.1	−0.01 (−0.11; 0.09)	−0.05 (−0.1; 0.10)
I am worried that my child might be assaulted when out alone in our neighbourhood.	6.83	**0.15 (0.02; 0.27)***	0.12 (−0.0; 0.30)
**4. Access to parks and playgrounds**: 5 point item (from −2 to +2):			
My child can play on a playground, park, or other public places (play street, schoolyard) in its neighbourhood without supervision.	82.9	−0.04 (−0.14; 0.07)	−0.10 (−0.2; 0.05)

* p-value ≤ 0.05; ** p-value ≤ 0.01; *** p-value ≤ 0.001. [1] All items had a five-point scale from strongly disagree (scored as -2) to strongly agree (scored as 2). [2] SB was transformed using the square root. [3] Adjustd for age, sex,household income, socioeconomic neighoburhood index (SEP),language region, urbanicity, season, accelerometer time and device model.

For road safety, aesthetics a higher score denotes less favorable environments and for personal safety and access to parks and playgrounds a higher score denotes a more favorable environment.

Table S2. Correlation between the perceived environment and the respective objective measures.

Perceived Environment	Objectively Assessed Environment	Spearman Correlation
**Road safety** (score −6 to 6))		
	Main street density (m/200m)	0.30
**Aesthetics and incivilities** (score −8 to 8)		
	Crime rate (n/100000 inhabitants)	0.19
**Personal safety** (score −10 to 10)		
	Walkability (z-score)	−0.04
	Cul de sac (n/200,)	0.05
	Number of school children (n/100m$^{2)}$	0.09
	Distance to the next bus stop (in m)	0.10
**Access to parks and playgrounds** (−2 to 2)		
	Green spaces (m^2/1000m radius)	0.09

For read safety, aesthetics and access to parks and playgrounds a higher score denotes less favorable environments and for personal safety a higher score denotes a more favorable environment.

Table S3. Mediation effects of the perceived environment on the associations between the objectively assessed environment and children's prolonged sedentary bouts on weekdays (square root transformation).

		Total Effect	Direct Effect	Indirect Effect			Significance of Mediation [1]
		c-path All values for *1000	c′-path All values for *1000	a-path All values for *1000	b-path	ab-path All values for *1000	
**Road safety**							
Main street density		0.0 (−0.3; 0.4)	0.0 (−0.4; 0.4)	**2.3 (1.8; 2.8) *****	0.02 (−0.02; 0.06)	0.04 (0.05)	-
**Aesthetics and incivilities**							
Crime rate		−0.9; (−4.3; 2.5)	−1.2 (−4.7; 2.3)	**10.8 (6.9; 14.7) *****	0.03 (−0.01; 0.08)	0.3 (0.3)	-
**Personal safety**							
Walkability		33.4 (−5.7.6; 72.6)	32.6 (−6.7; 71.8)	−43.4 (−108.8; 21.8)	−0.02 (−0.05;0.01)	0.9 (1.0)	-
Dead end		16.2 (−84.6; 117.0)	19.7 (−81.2; 120.6)	158.3 (−9.3; 325.9)	−0.02 (−0.05; 0.01)	−3.5 (3.2)	-
Number of school children		0.1 (−1.7; 1.8)	0.1 (−1.6; 1.8)	2.3 (−0.6; 5.2)	−0.02 (−0.05; 0.01	−0.1 (0.1)	-
Distance to the public transport		**−0.3 (−0.6; -0.0) ***	−0.3 (−0.6; −0.0)	0.3 (−0.0; 0.8)	−0.02 (−0.05; 0.01)	0.006 (0.007)	-
**Access to playgrounds**							
Green space (NDVI)	Score/1000m Buffer	**−1772.3 (−2949.3; −595.3) ****	−1760.2 (−2938.5; −581.8) *	450.9 (−1445.8; 1046.3)	−0.27 (−0.14; 0.08)	−12.1 (26.2)	Non-significant

* p-value ≤ 0.05, ** p-value ≤ 0.01, *** p-value ≤ 0.001. [1] The significance of the mediation was only tested if the total effect was statistically significant.

All anaylses adjustd for age, sex,household income, socioeconomic neighbourhood index,language region, urbanicity, season and accelerometer time

- c- coefficients: estimates of the associations between each item of the objective environment and children's time spent sedentary, e.g. main street density on time spent sedentary (square root transformation) on a weekday.
- c′-coefficients: estimates of the associations between the items of the objective environment and children's time spent sedentary, adjusted for the items of the perceived environment (mediator). e.g. main street density on time spent sedentary (square root transformation) on a weekday, adjusted for the road safety score for parental perceptions.
- a –coefficients: estimates of the associations between items of the objective environment and the items of the perceived environment (mediator), e.g. main street density and the read safety score for parental perceptions.
- b-coefficients: estimates of the associations between the items of the perceived environment (mediator) and children's time spent sedentary, adjusted for the items of the for the objective environment, e.g. the road safety score for parental perceptions on time spent sedentary (square root transformation) on a weekday, adjusted for the main street density.

The c′-path describes the direct effect of the objective environment on children's sedentary time, the a-path*b-path the possible indirect effect (see also fig. 1). Total effect c = c′ + a*b The total effect c= c′ + a*b

Table S4. Mediation effects of the perceived environment on the associations between the objectively assessed environment and children's prolonged sedentary bouts in the weekend.

		Total Effect:	Direct Effect:	Indirect Effect			Significance of Mediation [1]
		**c-path All values for *1000**	**c′-path All values for *1000**	**a-path All values for *1000**	**b-path**	**ab-path All values for *1000**	
**Road safety**							
Main street density	m/200m buffer)	0.0 (-0.5; 0.5)	0.0 (-0.5; 0.5)	**2.3 (1.8; 2.9)** ***	0.00 (-0.06; 0.06)	0.006 (0.06)	-
**Aesthetics and incivilities**							
Crime rate	n/100 000 inhabitants	-0.1 (-5.0; 4.9)	0.0 (-5.0; 5.0)	**11.1 (7.2; 14.9)** ***	-0.01 (0.08; 0.06)	-0.1 (0.4)	-
**Personal safety**							
Walkability	z-score (1000m)	-11.1(-67.8; 45.5)	-12.7 (-69.4; 43.9)	-44.7 (-109.9; 20.6)	-0.04 (-0.08; 0.01)	1.6 (1.6)	-
Dead end	n/200m buffer	-68.9 (-214.2; 76.5)	-63.3 (-208.8; 82.2)	160.4 (-7.0; 327.9)	-0.03(-0.08; 0.01)	-5.5 (4.8)	-
Number of school children	m/200 buf	0.2 (-2.3; 2.7)	0.3 (-2.2; 2.8)	2.2 (.0.6; 5.2)	-0.04 (-0.08; 0.01)	0.8 (0.8)	-
Distance to public transport		-0.1 (-0.5; 0.3)	-0.1 (-0.5; 0.3)	0.3 (-0.2; 0.8)	-0.04 (-82.7; 12.0)	-0.01 (0.01)	-
**Access to playgrounds**							
Green space	Score/1000 m Buffer	-708.7 (-2415.4; 998.1)	-664.5 (-2372.5; 1043.4)	455.5 (-139.9; 1051.9)	0.10 (-0.2; 0.06)	-44.1 (46.8)	-

* p-value ≤ 0.05, ** p-value ≤ 0.01, *** p-value ≤ 0.001. [a] The significance of the mediation was only tested if the total effect was statistically significant.

All anaylses adjustd for age, sex,household income, socioeconomic neighbourhood index,language region, urbanicity, season and accelerometer time

- c-coefficients: estimates of the associations between each item of the objective environment and children's time spent sedentary, e.g. main street density on time spent sedentary (square root transformation) on a weekday.

- c′-coefficients: estimates of the associations between the items of the objective environment and children’s time spent sedentary, adjusted for the items of the perceived environment (mediator). e.g. main street density on time spent sedentary (square root transformation) on a weekday, adjusted for the road safety score for parental perceptions.
- a–coefficients: estimates of the associations between items of the objective environment and the items of the perceived environment (mediator), e.g. main street density and the read safety score for parental perceptions.
- b-coefficients: estimates of the associations between the items of the perceived environment (mediator) and children’s time spent sedentary, adjusted for the items of the for the objective environment, e.g. the road safety score for parental perceptions on time spent sedentary (square root transformation) on a weekday, adjusted for the main street density.

The c′-path describes the direct effect of the objective environment on children’s sedentary time, the a-path*b-path the possible indirect effect (see also fig. 1). Total effect $c = c' + a*b$ The total effect $c = c' + a*b$.
